# Median Rhomboid Glossitis of the Tongue-Associated Kissing Lesion: A Report of a Rare Case

**DOI:** 10.7759/cureus.65419

**Published:** 2024-07-26

**Authors:** Sourabh B Shinde, Vidya Lohe, Swapnil Mohod, Komal V Dadgal, Madhu Priya, Tikeshwari Gurav

**Affiliations:** 1 Department of Oral Medicine and Radiology, Sharad Pawar Dental College and Hospital, Datta Meghe Institute of Higher Education and Research, Wardha, IND; 2 Department of Prosthodontics, Sharad Pawar Dental College and Hospital, Datta Meghe Institute of Higher Education and Research, Wardha, IND; 3 Department of Endodontics, Sharad Pawar Dental College and Hospital, Datta Meghe Institute of Higher Education and Research, Wardha, IND

**Keywords:** lesions on dorsum of tongue, oral candidiasis, lesions on palate, kissing lesion, median rhomboid glossitis

## Abstract

A 50-year-old female patient visited the dental outpatient department with the chief complaint of ulceration associated with pain and burning sensation on the anterior and middle parts of the tongue and the posterior portion of the hard palate, which showed contact lesions that were similar in shape and size. The patient gave the history of the following symptoms a week before the patient visited the department. The patient was asymptomatic six months ago when she initially observed yellowish deposits on her tongue that could be scraped off. The patient misjudged these deposits as food debris and did not undergo any treatment for them. It was in the past week that she developed ulceration on the posterior portion of the hard palate.

## Introduction

Disturbances of growth and development resulting in anomalies are often referred to as developmental anomalies, which can be congenital, i.e., acquired through the genes. Median rhomboid glossitis is one such anomaly that is associated with the congenital abnormality caused by diminished growth of tuberculum impar. In the early literature, the condition was termed “Glossite Lasangigue Mediane De La Face Dorsal Langue.” It was because of the appearance of the lesion as benign in the midline of the dorsum of the tongue lying posteriorly and at the middle third, just in front of the circumvallate papillae [1]. The lesion is usually rhomboid but can be oval, circular, or elliptical. Its characteristic features include depapillation of the tongue, especially in the midline, just anterior to the circumvallate papillae. Previously, it was considered to be due to over-retaining of tuberculum impar, but now it falls under the category of lesions caused by Candida infection [1,2]. It is a rare condition that occurs in between 0.01% and 0.1% of individuals, and it is seen more commonly in males as compared to females [2]. It is asymptomatic, which is why it goes unnoticed until the middle ages of the individuals involved [2].

Human immunodeficiency virus (HIV) can be one of the reasons for the median rhomboid glossitis and kissing lesion. Although there is not enough literature to support that it affects directly, it can create an environment for opportunistic candidal infection to cause the lesion. As HIV brings an immunocompromised state, it can facilitate Candida infections, leading to median rhomboid glossitis [3]. Candidiasis is caused by *Candida albicans* (*C. albicans),* a fungal organism with a yeast-like appearance. The infection usually presents itself in one of four forms, namely, hyperplastic candidiasis, erythematous candidiasis, pseudomembranous candidiasis, or angular cheilitis [3,4]. Candidiasis is one of the earliest manifestations of HIV; one can also suspect median rhomboid glossitis and kissing lesions associated with cases of HIV [4]. Another reason that does not affect it directly but can create an environment that facilitates the growth of Candida and leads to kissing lesions is the use of cannabis and cannabis products [5].

## Case presentation

A 50-year-old female patient visited the dental outpatient department with the chief complaint of ulceration associated with pain and burning sensations on the anterior and middle parts of the tongue and the posterior portion of the hard palate. The patient gave the history of the following symptoms a week before she visited the department. The patient was asymptomatic six months ago, and then she noticed whitish deposits on her tongue for the first time, which were scrapable. The patient considered it as food debris and did not undergo any treatment for the deposits. In the past week, she has developed an ulcer on the posterior portion of the hard palate. The patient also gave a history of an increase in the size of the ulcer with an increased intensity of pain as compared to the first incidence. The tongue showed depapillation and ulceration on the mid portion of the dorsal surface, which was covered by erythema and a scrapable white candidal lesion. The Visual Analogue Score (VAS) was 10 on a scale of 10, with 0 being no pain and 10 being unbearable pain. The patient gave a history of dehydration one week ago with no recovery to date.

The patient additionally reported a decreased consumption of food and liquids due to a burning sensation. There was a history of bleeding from the site of the ulceration on the tongue two to three days ago. The past medical history of the patient stated that she had undergone surgery in the left ear due to a fungal infection two years ago, with a history of recurrence of the infection one year later. There was no history of any current infection, nor was there any infection present. She is a known case of hypertension and has been on medications for the same for 10 years (Tab. Telmisartan 40 mg once daily morning dose). The patient also had a history of varicose vein thrombosis for two years and was on treatment for it.

**Figure 1:**
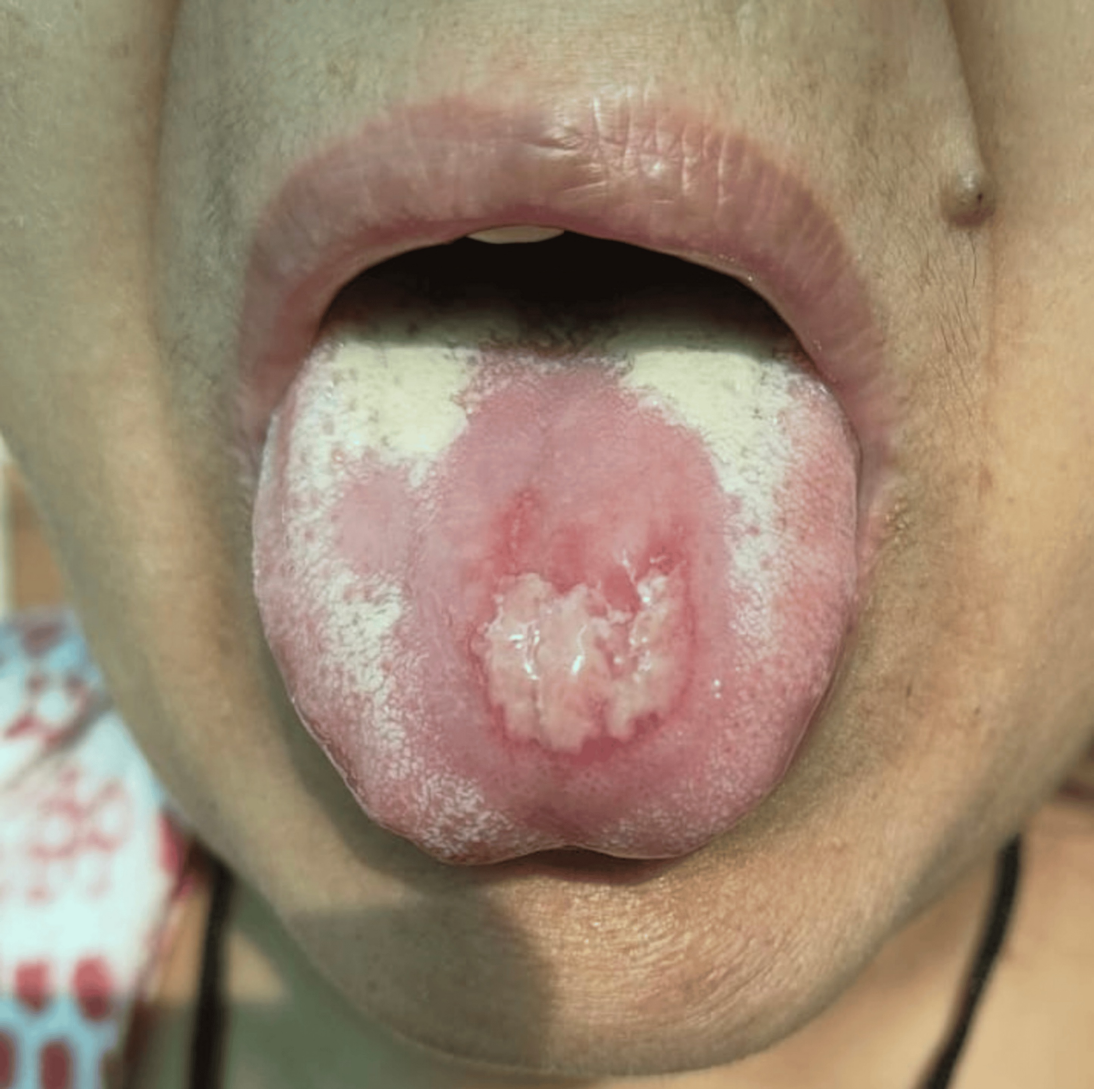
The depapillation area, followed by the lesion on the dorsal surface of the tongue.

Furthermore, the patient reported a history of root canal treatment one year ago. She did not report any history of deleterious habits, nor was she allergic to any known medications to date. The extra oral features were normal, with no asymmetry associated with the face or the lips. The temporomandibular joint did not show any abnormalities. Bilateral submandibular lymphadenopathy was present with a single node of size 0.8 × 0.08 cm, approximately palpable in the left as well as the right submandibular region. The nodes were soft, mobile, and tender (VAS: 4-5). On intra-oral examination, there was an area of depapillation in the mid portion of the dorsal surface of the tongue. The area contained a lesion that was rhomboid in shape with well-defined borders that were approximately 1 × 1 cm in size. This presentation is characteristic of median rhomboid glossitis (Figure 1).

**Figure 2:**
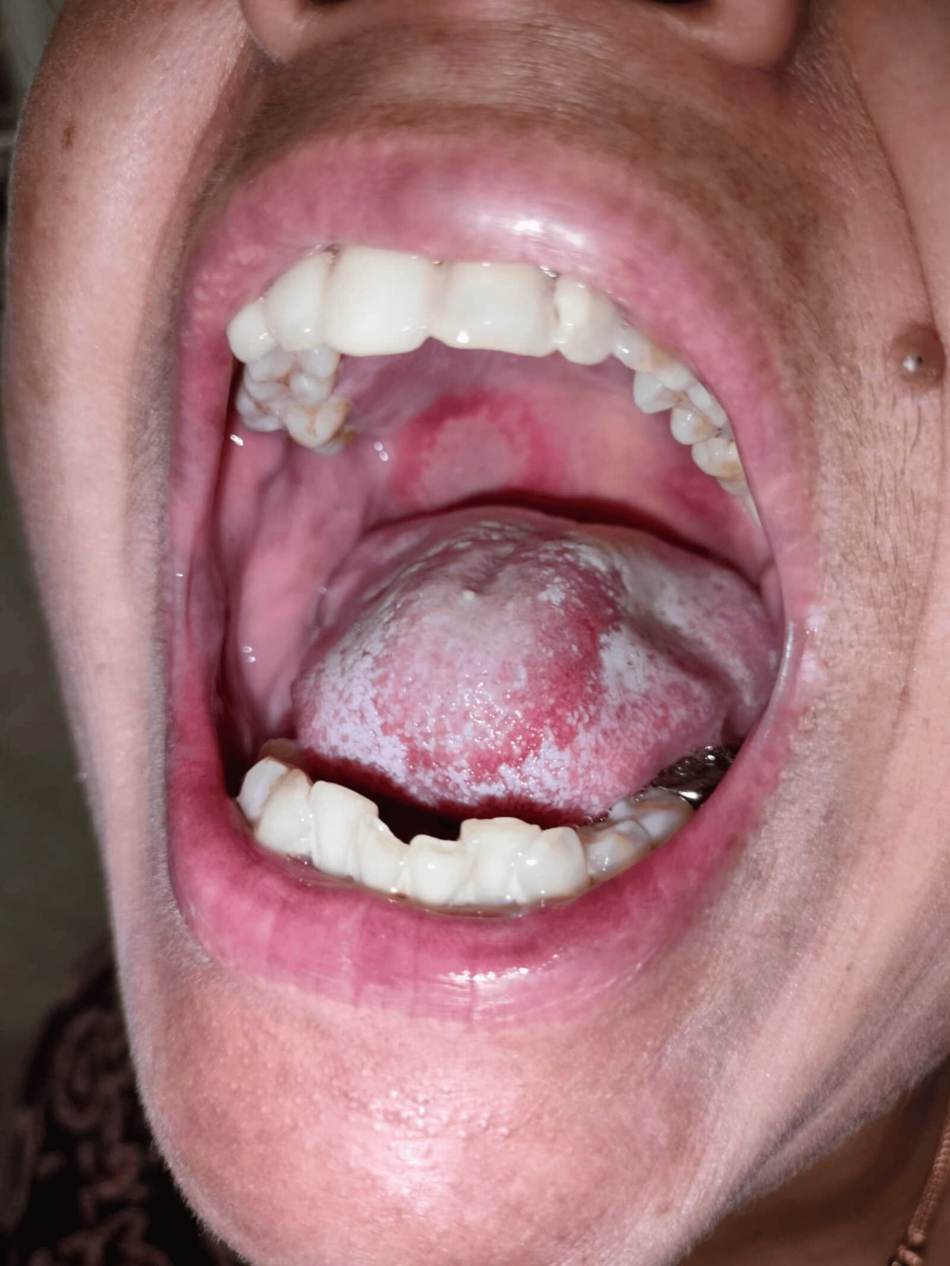
Ulcer on the posterior portion of the hard palate.

The ulcer was oval with well-defined borders and showed a reddish periphery with central erythema. A similar ulcer was seen on the posterior portion of the soft palate, as shown in Figure 2.

**Figure 3:**
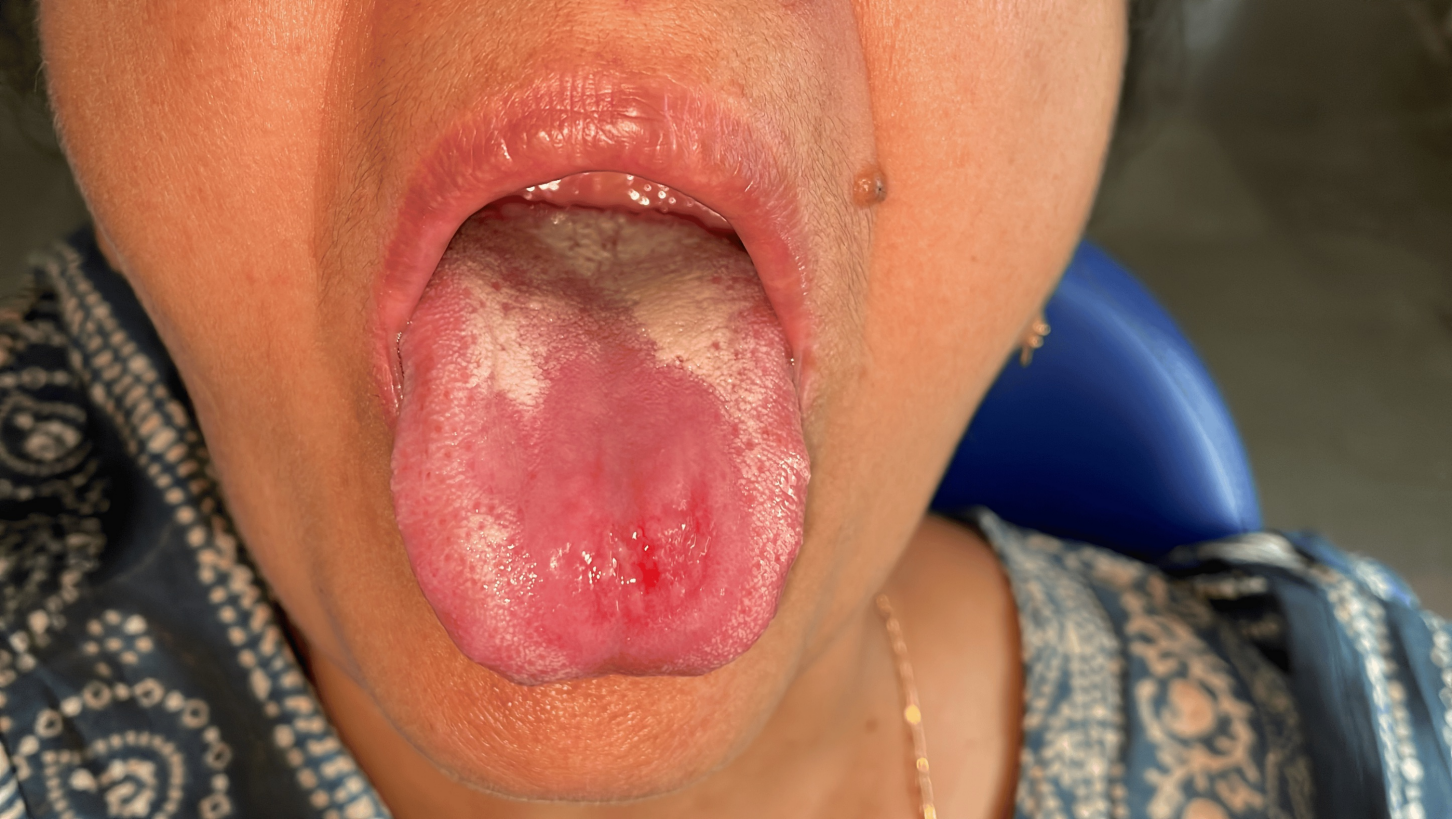
Reduced candidal infection and reduction in size of the ulcer.

**Figure 4:**
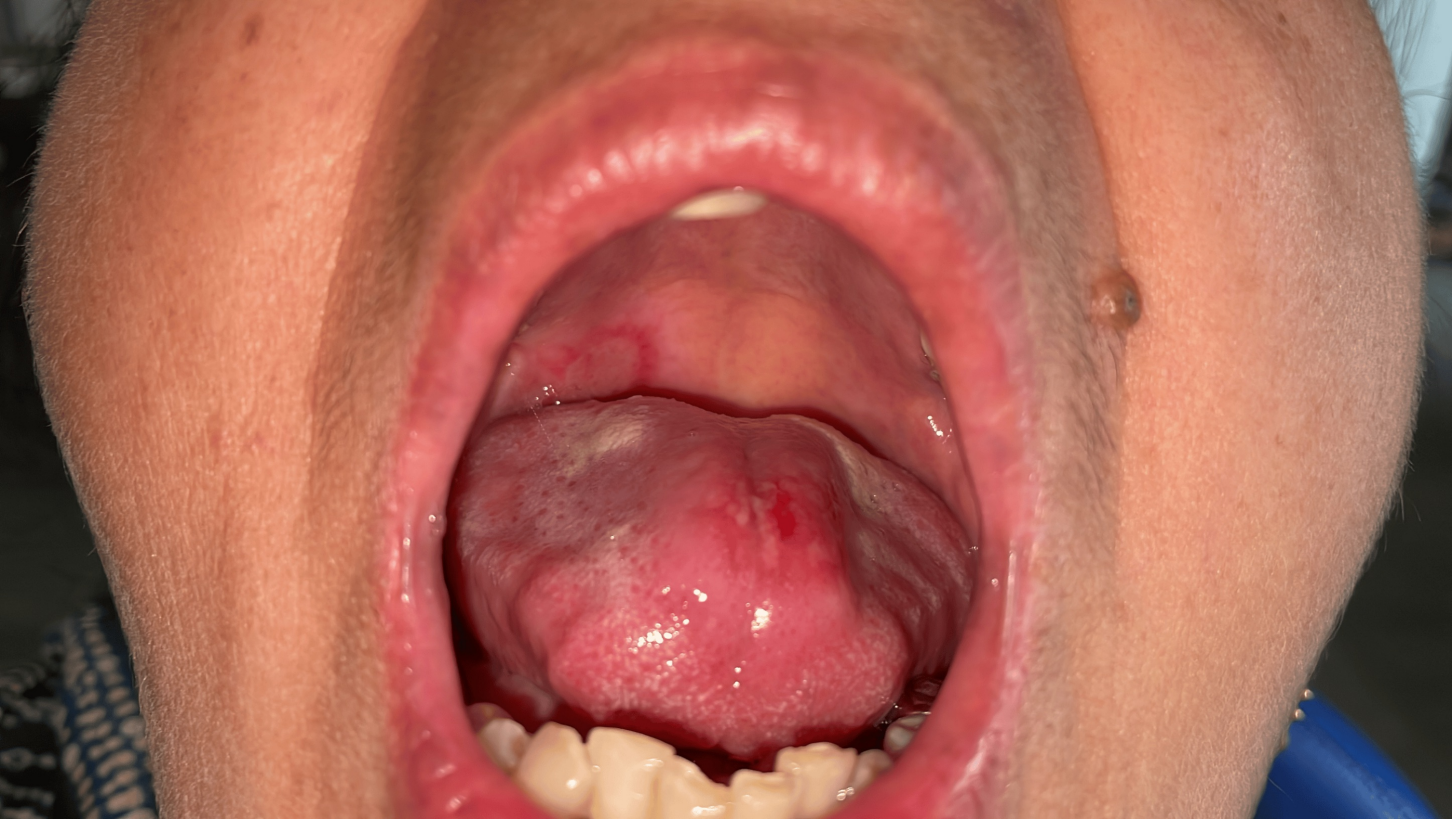
Reduction in the size and severity of the palatal ulcer.

The clinical provisional diagnosis was given as a median rhomboid glossitis-associated kissing lesion on the dorsal surface of the tongue and posterior portion of the soft palate. The patient was advised to take tablet AF 150 (Fluconazole dispersible tablet 150 mg) and was advised to take two tablets dissolved in water for oral uptake. Then, the next two tablets were scheduled after one week, and another two tablets after another one week. This was followed by daily application of candid mouth paint (Clotrimazole Mouth Paint) four times daily for two weeks and tablet Fibrowin (myoinositol, L-tyrosine, iron, magnesium, vitamin B7, vitamin B12, vitamin D3, vitamin B3, vitamin B5, vitamin B6, folic acid, chromium picolinate, selenium, and zinc) once daily at night for two weeks for multivitamin and antioxidant therapy. The patient was recalled after two weeks for review. On the second visit, the patient reported experiencing 50% relief from all the previous symptoms. Her intra-oral examination also showed healing of ulceration and a subsequent reduction in the severity of the candidal lesion (Figures 3, 4).

**Figure 5:**
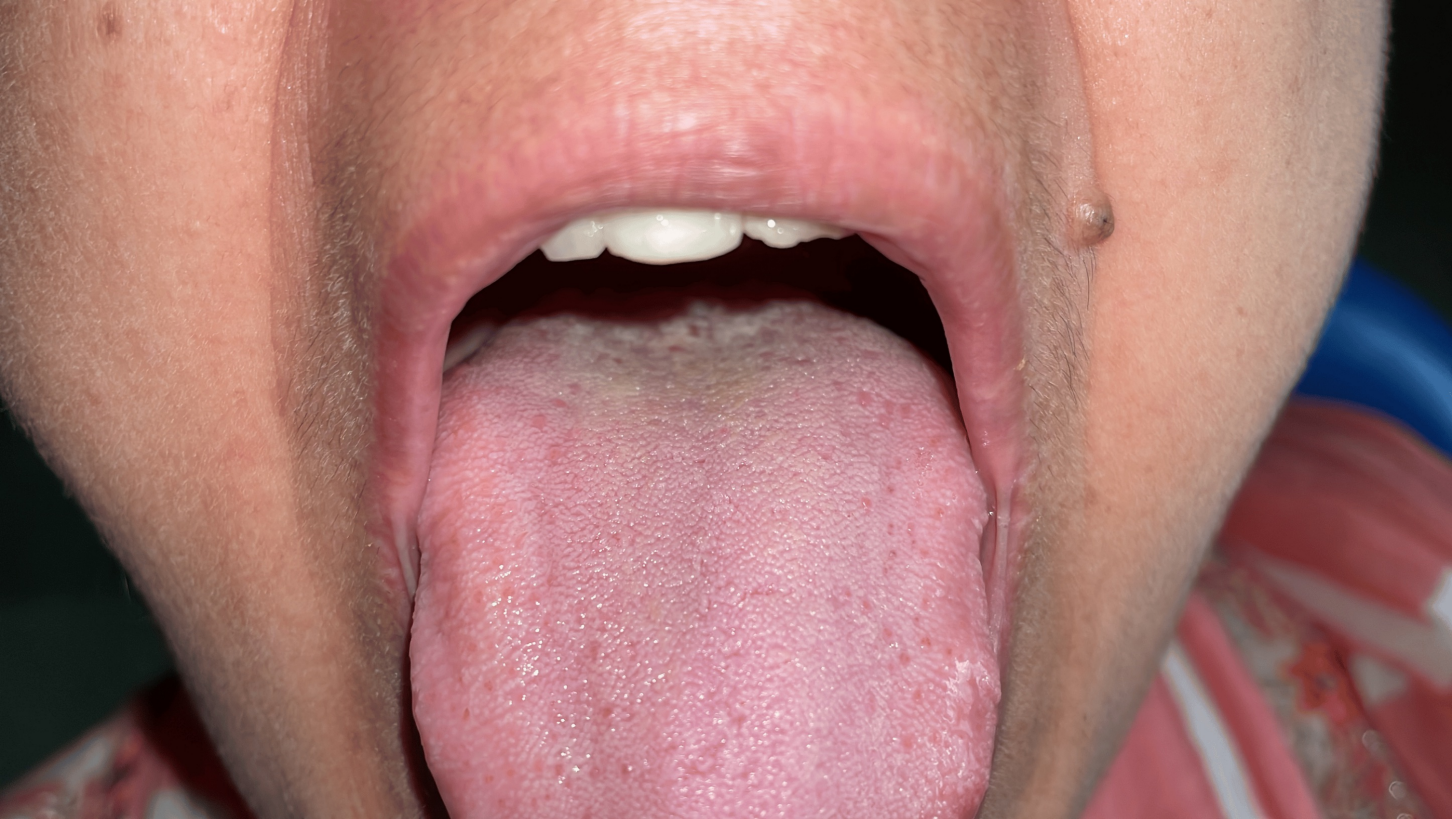
Complete healing of the candidal lesion and the ulceration.

**Figure 6:**
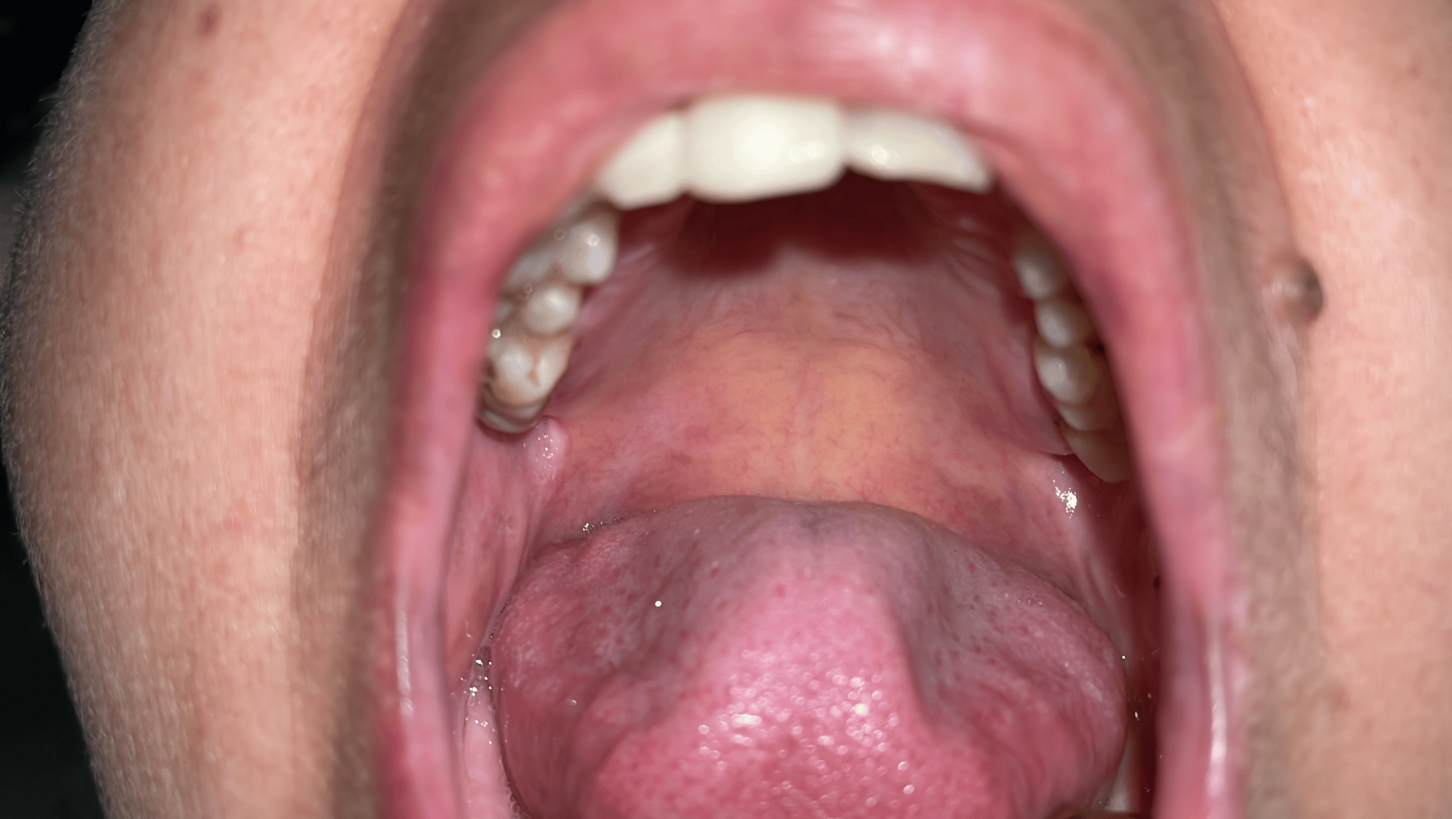
Complete healing of the palatal ulceration.

The intensity of pain and burning sensation was reduced from VAS 10 to VAS 4. The patient was able to intake the liquids but was on a diet of bland and less spicy foods. The patient also reported improved hydration. All the medications were again continued for two more weeks at a similar dosage and time, and the patient was recalled after two weeks for review. On the third visit, the patient reported complete recovery from pain and burning sensations with a VAS of 0. She was able to intake all kinds of foods and liquids and completely recover from dehydration. Upon intra-oral examination, it was observed that the lesion on the tongue and palate had completely healed, including the candidal lesion (Figures 5, 6).

The patient's prescription for tablet Fibrowin was extended for an additional month, while tablet AF 150 and Candid mouth paint were discontinued.

## Discussion

Some studies suggest that smoking plays a key role in developing candidiasis and subsequent kissing lesions. According to the literature provided by Goregen et al., there have been some studies performed on diabetic and non-diabetic individuals who were smokers. The studies concluded that the candidal load was higher in patients with a history of smoking than in healthy individuals [6]. In our patient, there was no history of smoking or any tobacco habits. A study by Elmezwghi et al. conducted on the incidence of diabetic and non-diabetic individuals concluded that the prevalence in diabetic patients was higher than that of non-diabetic individuals (Table 1) [7].

**Table 1:** Association of age and diabetes mellitus with median rhomboid glossitis, according to Elmezwghi et al.

Age Groups	Lesions in Diabetic-Controlled Patients	Lesions in Diabetic-Uncontrolled Patients	Total
18 to 25	4	8	12
26 to 33	16	12	18
34 to 41	13	33	46
42 to 49	25	44	69
50 to 58	39	88	127
59 to 66	31	99	130
67 to 74	19	29	48
75 to 82	3	9	12
More than 82	1	1	2
Total	141	323	464

It also found that there was an increased burning sensation in patients with diabetes than the non-diabetic individuals. It suggested that if a patient with median rhomboid glossitis comes with a complaint of a burning sensation, they should be assessed for diabetes mellitus [7]. In our patient, due to the presence of a burning sensation, we advised her to undergo HbA1C and random blood sugar tests. However, the patient declined to take the tests. There have been certain cases where the median rhomboid glossitis was a part of vigorous tongue brushing. According to the literature provided by Shindo, there have been incidents when median rhomboid glossitis was caused by the patient's aggressive brushing practices. When a sample from the affected area was examined for candidal infection, the results were negative [8].

In recent decades, azole agents have been preferred for the management of fungal infections. Fluconazole, being the most common azole agent that is used for the treatment of fungal infections worldwide, has undergone resistance due to overuse, particularly in fluconazole-resistant Candida spp. [9]. Similarly, in the field of biomedicine, the emergence of DNA sequencing has promised a precise and accurate approach to the treatment of fungal infections. However, there is still a need for precise diagnosis from clinicians to reduce the inappropriate use of antifungal agents [9].

## Conclusions

Previously believed to be a developmental defect, median rhomboid glossitis is not considered under the embryogenesis theory by most of the authors. Now the relation of the lesion relates to the Candida and associated infections. When there is an associated kissing lesion with median rhomboid glossitis, immunosuppression is the major suspect. Hence, a proper diagnosis and a well-designed treatment plan are necessary when it comes to median rhomboid glossitis-associated kissing lesions. As in the above-discussed case, there was a positive history of fungal infection, associated surgery, and recurrence. The patient presented with a burning sensation, so we advised an HbA1C test. For the treatment protocol, we advised antifungal medications and supportive therapy with antioxidants and multivitamins to replenish the loss due to dehydration. Due to its rarity, understanding median rhomboid glossitis can be difficult, so the medical history of the patient and general condition, along with lifestyle, play a major role. Therefore, a comprehensive evaluation of the history, general condition, and lifestyle is necessary.
